# Effectiveness of a nurse practitioner-led cardiovascular prevention clinic at reduction of metabolic syndrome following maternal complications of pregnancy: a preliminary analysis

**DOI:** 10.1186/s13098-022-00916-8

**Published:** 2022-10-06

**Authors:** Emily Aldridge, Maleesa Pathirana, Melanie Wittwer, Susan Sierp, Shalem Y. Leemaqz, Claire T. Roberts, Gustaaf A. Dekker, Margaret A. Arstall

**Affiliations:** 1 Adelaide Medical School, University of Adelaide, Adelaide, South Australia Australia; 2 Robinson Research Institute, University of Adelaide, Haydown Road, Elizabeth Vale, Adelaide, South Australia Australia; 3 Department of Cardiology, Northern Adelaide Local Health Network, Elizabeth Vale, Adelaide, South Australia Australia; 4 Flinders Health and Medical Research Institute, Flinders University, Bedford Park, South Australia Australia; 5 Department of Obstetrics and Gynaecology, Northern Adelaide Local Health Network, Elizabeth Vale, Adelaide, South Australia Australia

**Keywords:** Metabolic syndrome, Cardiovascular disease prevention, Pregnancy complications, Maternal health

## Abstract

**Aim:**

Maternal complications of pregnancy, including hypertensive disorders of pregnancy, gestational diabetes mellitus, intrauterine growth restriction, preterm labour, and placental abruption, are associated with increased risk of future cardiometabolic disease. Lifestyle interventions that focus on preventative strategies for this young, high-risk population of women may assist in cardiometabolic disease risk reduction. The aim of this preliminary registry analysis was to observe the change in maternal metabolic syndrome status after receiving a nurse practitioner-led lifestyle intervention delivered soon after a complicated pregnancy.

**Method:**

This preliminary analysis included 64 eligible women who had attended both baseline (approximately 6 months postpartum) and review (approximately eighteen months postpartum) appointments at the postpartum lifestyle clinic after an index pregnancy complicated by at least one maternal complication of pregnancy. Metabolic syndrome status at both appointments was assessed.

**Results:**

At the baseline appointment, 22 (34.4%) women met the criteria for metabolic syndrome. This number reduced at the review appointment to 19 (29.7%). This difference was not statistically significant. There were some modest improvements in the individual cardiometabolic risk factors, as well as marked improvements in the women who had recovered from metabolic syndrome over twelve months.

**Conclusion:**

There was a high percentage of metabolic syndrome present early in the postpartum period. The results of this preliminary analysis highlight the importance of continuing preventative care and ongoing research for this group of high-risk women.

## Introduction

Certain complications of pregnancy, including hypertensive disorders of pregnancy, gestational diabetes mellitus, intrauterine growth restriction, spontaneous preterm labour, and placental abruption, affect one quarter of all pregnancies in Australia [1] and are associated with a significantly increased maternal risk of cardiovascular and metabolic disease in later life [2–10]. Pregnancy provides a unique opportunity to identify young women at risk of cardiometabolic disease, and the early postpartum period is an ideal time to introduce primary prevention strategies as women are already engaged with the healthcare system.

Recommendations for monitoring women with a previous history of a complication of pregnancy have been published for over a decade [11, 12], yet availability of these services and patient uptake remains relatively low. The first and most notable standardised postpartum prevention clinical model is the Maternal Health Clinic in Kingston, Canada [13] which, since its inception in 2011, has been replicated in other locations across Canada and the world. An analysis of clinic attendance between pregnancies and subsequent pregnancy outcomes found that attendance at the Maternal Health Clinic was associated with lower inter-pregnancy weight gain and fewer diagnoses of type 2 diabetes [14]. However, the true effectiveness of this clinic at reducing cardiovascular risk long-term has yet to be investigated. In Australia, there are very few postpartum follow-up services for this high-risk cohort. In 2018, the first nurse-practitioner led postpartum lifestyle clinic was introduced at a metropolitan hospital based in the northern suburbs of Adelaide, South Australia [15]. This outpatient service was developed using the Maternal Health Clinic clinical model and provides cardiometabolic risk screening and individualised lifestyle and risk reduction education for all women who experience a serious complication of pregnancy. The effectiveness of this clinic is also yet to be assessed.

The effectiveness of lifestyle interventions at reducing cardiometabolic risk in women following a complicated pregnancy has not been thoroughly explored, primarily due to the fact that these services are relatively new and target a young population of women who have not yet reached an age where cardiovascular events would be expected to occur. The true long-term effectiveness of postpartum preventative strategies at reducing the incidence of major adverse cardiovascular events may therefore remain unknown for some years to come. Accurately assessing cardiovascular and metabolic risk in younger women in general is made difficult by ineffective traditional cardiovascular risk calculators which underestimate absolute risk in women and young people, and do not take into account pregnancy history [16].

Metabolic syndrome, a cluster of the most dangerous heart attack risk factors, is associated with a 4-fold increased risk of developing cardiovascular disease in women [17] and is a preclinical marker of risk in younger patients. Recovery from metabolic syndrome is significantly associated with a decreased risk for major adverse cardiovascular events, providing clear clinical targets for improvement [18]. Metabolic syndrome is detectable in younger cohorts and may therefore be a useful surrogate for absolute cardiovascular risk in young women, and has been assessed in a number of previous studies of maternal health following pregnancy complications [19–24]. In a preliminary analysis of our cohort at 6 months postpartum, metabolic syndrome was present in 36% of women [25].

The aim of this study was to assess the percentage change in metabolic syndrome over time in a cohort of women with a history of a complication of pregnancy who attended the postpartum lifestyle clinic. It was hypothesised that there would be a lower overall percentage of metabolic syndrome at approximately eighteen months postpartum review compared with that at 6 months postpartum.

## Methods

### Study design and setting

This was a prospective observational study of the postpartum lifestyle clinic at the Lyell McEwin Hospital (LMH) [15]. The LMH is a public tertiary acute-care facility providing obstetric care, adult cardiac and intensive care services, and neonatal care for infants at ≥ 32 weeks’ gestation and located within the Northern Adelaide Local Health Network (NALHN), South Australia. The NALHN area is characterised by a population with low socioeconomic status with high rates of CVD morbidity and mortality, and is among Australia’s most disadvantaged suburban communities [26]. The Central Adelaide Local Health Network Human Research Ethics Committee approved the study [HREC/16/TQEH/258].

### The postpartum lifestyle intervention clinic

Methods for the hospital-based outpatient clinical service and associated quality assurance registry have been previously described [15]. Briefly, to be eligible for referral to the postpartum lifestyle clinic, women must have experienced at least one of the following complications in their index pregnancy: hypertensive disorder of pregnancy requiring medical therapy or resulting in birth < 37 weeks’ gestation, gestational diabetes mellitus requiring metformin or insulin therapy, spontaneous preterm labour < 34 weeks’ gestation, intrauterine growth restriction or delivery of a small for gestational age infant at < 5th customised birth centile, or placental abruption. At approximately 6 months postpartum, participants undergo a thorough baseline health assessment and receive individualised health counselling from an expert nurse practitioner. Variables are collected from a combination of patient self-report and abstraction from the hospital medical record. Information collated and included in the registry includes patient demographics, medical history, family history, current medications, alcohol, drug and smoking practices, obstetric history, cardiovascular and metabolic screening pathology results, systolic and diastolic blood pressures, height, weight, and waist circumference. Blood pressure was measured using an oscillometric pulse wave analysis device, the USCOM BP + [USCOM, Sydney, Australia]. This device has been previously validated against aneroid blood pressure measurements [27]. A review appointment is conducted at 18 months postpartum where all study measures are repeated.

Following collection of the clinical and demographic information, the nurse practitioner provides individualised health and lifestyle education and clinical counselling aiming to improve modifiable cardiovascular risk factors. General dietary advice based on the Australian Heart Foundation Heart Healthy Eating Patterns position statement and Australian Dietary Guidelines is provided [28, 29]. Broadly, these guidelines recommend eating plenty of fruit, vegetables and whole grains, a variety of heart-healthy protein sources and limiting the intake of red meat, consuming healthy dairy choices (and choosing reduced-fat dairy if blood cholesterol is high), choosing healthy fat sources, and using herbs and spices in the place of salt. Physical activity advice is provided to women in alignment with *Australia’s Physical Activity and Sedentary Behaviour Guidelines (18–65 years)* and the Australian Heart Foundation exercise guidelines [30, 31], which recommend at least 30 min of daily exercise and incorporating a combination of aerobic and muscle-strengthening exercises. Additional investigations may be ordered where necessary, and referrals to medical specialists or other services are made where appropriate. A formal report summarising patient results are forwarded to the nominated general practitioner.

### Study inclusion/exclusion criteria

Women who attended both the baseline and review appointments at the postpartum clinic from 7th August 2018 to 14th July 2021 were included. Women who were pregnant at either baseline or review appointment, did not complete pathology testing, or did not attend their review appointment, were excluded as metabolic syndrome status could not be accurately determined.

### Outcomes

The primary outcome of interest for this study was the reduction in percentage of metabolic syndrome from baseline to review. This change in percentage was considered a surrogate measure for the effectiveness of the intervention delivered at baseline.

Metabolic syndrome was defined as the presence of any 3 of the following five risk factors [32]:

- Elevated waist circumference with ethnicity specific values defined by the International Diabetes Federation [33], which for women is ≥ 80 cm for all ethnicities
- Elevated triglycerides of ≥ 1.7 mmol/L, or drug treatment for this lipid abnormality
- Reduced HDL cholesterol of < 1.3 mmol/L, or drug treatment for this lipid abnormality
- Elevated systolic blood pressure of ≥ 130 mmHg and/or diastolic blood pressure of ≥ 85 mmHg, or antihypertensive drug treatment
- Elevated fasting glucose of ≥ 5.6 mmol/L, or drug treatment of elevated glucose.

Secondary outcomes in this study included individual cardiovascular and metabolic risk factors, such as waist circumference, body mass index, peripheral systolic and diastolic blood pressures, triglycerides, HDL cholesterol, fasting plasma glucose, fasting insulin, and smoking status.

### Analysis

Continuous variables are presented as mean and standard deviation and categorical data are presented as count and percentage. Values were rounded to the nearest 2 decimal places. McNemar’s test was conducted to assess for the difference between metabolic syndrome status at the 2 time points. Paired t-tests were used to assess the mean differences in continuous variables between the 2 time points. The change in metabolic syndrome status from baseline to review was categorised into 4 groups (no metabolic syndrome at either timepoint, metabolic syndrome at both timepoints, metabolic syndrome at first timepoint but not at second timepoint, and no metabolic syndrome at first timepoint but development of metabolic syndrome at second timepoint), and descriptive statistics of the change in cardiometabolic risk factors for each group were reported. Further analysis to assess the risk of metabolic syndrome status at review was undertaken using binary logistic regression with backwards stepwise elimination, adjusting for metabolic syndrome status at baseline, age, parity, ethnicity and socioeconomic status. All analyses were conducted using IBM SPSS Statistics for Windows, version 28.0 (Armonk, NY: IBM Corp).

### Results

**Fig. 1:**
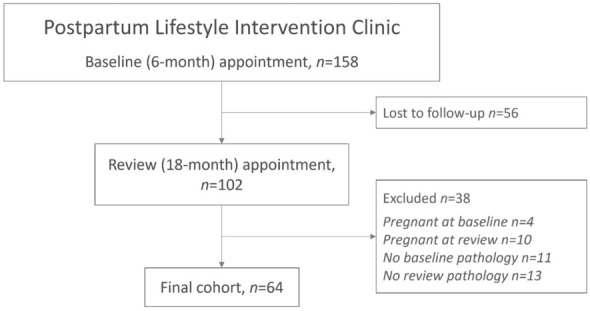
Study inclusion flow-chart

The number and proportion of eligible women included in this study was 64 (40.5%) out of a possible 158. The inclusion and participation information is displayed in Fig. 1. The average time from referral to the first appointment was 7.5 ± 1.9 months, and the average time between the first appointment and the review appointment was 14 ± 3.9 months. None of the women had an intervening pregnancy between the appointments.

Participant demographics at the time of first appointment are displayed in Table 1. Majority of participants were married or in de facto relationships (90.7%), spoke English as their preferred language (81.2%) and had experienced gestational diabetes (68.8%) in their index pregnancy. The mean age of the cohort was 33 years. The average number of children for each participant was 2, and the range of parity was 1–6.

**Table 1:** Participant demographics at baseline (6 months postpartum), n = 64

Participant characteristics	Mean ± standard deviation, or *n* (%)
Age, years	33.08 ± 5.75
Gravidity	2.80 ± 1.65
Parity	2.20 ± 1.34
Index pregnancy booking BMI, kg/m^2^	29.04 ± 7.00
SEIFA IRSAD	922.56 ± 57.50
White	35 (54.7)
Referring pregnancy complication	
HDP	24 (37.5)
GDM	44 (68.8)
IUGR	5 (7.8)
PTL	0
PA	0
Required interpreter	12 (18.8)
Marital status	
Married	44 (68.8)
De facto	14 (21.9)
Separated	1 (1.6)
Single	5 (7.8)
Employment	
Unemployed	28 (43.8)
Maternity leave	16 (25.0)
Casual	10 (15.6)
Part-time	9 (14.1)
Full-time	1 (1.6)
Medicated hypertension	4 (6.3)
T1DM	2 (3.1)
T2DM	1 (1.6)
Current smoking	6 (9.4)
Current illicit drug use	1 (1.6)

*BMI* body mass index, *SEIFA IRSAD* Socioeconomic Index for Area Index of Relative Socioeconomic Advantage and Disadvantage, *HDP* hypertensive disorders of pregnancy, *GDM* gestational diabetes mellitus, *IUGR* intrauterine growth restriction, *PTL* preterm labour, *PA* placental abruption, *T1DM* type 1 diabetes mellitus, *T2DM* type 2 diabetes mellitus

Table 2 shows metabolic syndrome status and cardiometabolic risk factors for the cohort at both baseline and follow-up. There were no statistically significant differences between time points, although there were minor reductions in the percentage of the cohort with metabolic syndrome and mean waist circumference, systolic blood pressure, lipids, glucose, and insulin at review.

**Table 2:** Metabolic syndrome status and risk factors at baseline and review, n = 64

	Baseline	Review	p-value
Metabolic syndrome present, n (%)	22 (34.4)	19 (29.7)	0.58
Number of metabolic syndrome criteria present			
0	6 (9.4)	7 (10.9)	
1	16 (25.0)	16 (25.0)	
2	19 (29.7)	22 (34.4)	
3	14 (21.9)	11 (17.2)	
4	8 (12.5)	7 (10.9)	
5	0 (0)	1 (1.6)	
BMI, kg/m^2^	30.27 ± 7.09	30.40 ± 7.38	0.31
Waist circumference, cm	95.24 ± 14.99	95.11 ± 14.56	0.23
Peripheral SBP, mmHg	122.22 ± 13.12	120.97 ± 12.73	0.11
Peripheral DBP, mmHg	73.09 ± 10.57	73.11 ± 11.67	0.49
Triglycerides, mmol/L	1.37 ± 0.93	1.27 ± 0.71	0.18
HDL cholesterol, mmol/L	1.28 ± 0.28	1.25 ± 0.30	0.18
Glucose, mmol/L	5.20 ± 1.20	5.15 ± 0.77	0.44
Insulin, mU/L	15.22 ± 15.54	12.22 ± 7.69	0.09
HOMA-IR	3.54 ± 3.72	2.84 ± 1.99	0.24
Medicated hypertension	4 (6.3)	6 (9.4)	
Medicated T2DM	1 (1.6)	2 (3.1)	
Current smoking	6 (9.4)	5 (7.8)	

*BMI* body mass index, *SBP* systolic blood pressure, *DBP* diastolic blood pressure, *HDL* high-density lipoprotein, *HOMA-IR* Homeostatic Model Assessment for Insulin Resistance, *T2DM* type 2 diabetes mellitus

Looking more in depth at the change in metabolic syndrome status over time, 79.7% of the cohort presented with the same metabolic syndrome status at review as baseline (no metabolic syndrome at either timepoint, 57.8%; metabolic syndrome at both timepoints, 21.9%). Of note, 12.5% of the cohort had metabolic syndrome at baseline but no longer met the criteria at review; conversely, 7.8% did not have metabolic syndrome at baseline but had developed it by the time of review. The mean changes in cardiometabolic risk factors are presented in Table 3.

**Table 3:** Mean change in cardiometabolic risk factors over time

	No MetS at either time point, n = 37	MetS at first timepoint, no MetS at second timepoint (MetS resolved), n = 8	No MetS at either timepoint, n = 14	Developed MetS during study: No MetS at first timepoint, MetS at second timepoint, n = 5
BMI, kg/m^2^	0.31 ± 2.40	− 0.87 ± 2.63	0.29 ± 1.49	0.08 ± 1.42
Waist circumference, cm	− 0.21 ± 12.88	− 3.71 ± 4.22	0.39 ± 5.52	− 0.02 ± 2.94
Peripheral SBP, mmHg	− 4.57 ± 19.90	− 3.12 ± 8.22	− 1.64 ± 11.00	3.20 ± 6.65
Peripheral DBP, mmHg	− 2.54 ± 14.67	− 1.38 ± 9.97	1.21 ± 8.67	3.20 ± 9.26
Triglycerides, mmol/L	− 0.09 ± 0.78	− 0.45 ± 0.67	0.12 ± 0.77	0.32 ± 0.25
HDL cholesterol, mmol/L	0.54 ± 0.41	0.10 ± 0.19	− 0.50 ± 0.13	0.40 ± 0.27
Glucose, mmol/L	0.22 ± 1.97	− 0.20 ± 1.21	0.41 ± 1.80	0.44 ± 0.89
Insulin, mU/L	− 1.44 ± 16.16	− 12.58 ± 16.44	1.10 ± 10.69	− 0.42 ± 8.26
HOMA-IR	− 0.39 ± 3.66	− 3.26 ± 5.02	0.34 ± 3.56	0.15 ± 2.32

Data are presented as mean ± standard deviation. ‘Improve’ denotes those who had MetS at the first time point but did not meet the criteria at the second time point. ‘No change’ denotes those who had MetS at both time points. ‘Worse’ denotes those who did not have MetS at the first time point but met the criteria at the second timepoint

*MetS* metabolic syndrome, *BMI* body mass index, *SBP* systolic blood pressure, *DBP* diastolic blood pressure, *HDL* high-density lipoprotein, *HOMA-IR* Homeostatic Model Assessment for Insulin Resistance

A binary logistic regression was conducted with backwards stepwise elimination to further explore the risk of metabolic syndrome at review, adjusting for metabolic syndrome status at baseline, age, parity, ethnicity, and socioeconomic status. Based on the probability criteria (*F*-to-enter = 0.05 and *F*-to-remove = 0.10), ethnicity and socioeconomic status were eliminated from the model, with age and parity remaining as variables of interest. For every additional year in age, there was a 12.7% increase in odds of meeting metabolic syndrome criteria at review (adjusted odds ratio (aOR): 1.13, 95% confidence interval (CI) 0.98, 1.30; p = 0.10), although this was not statistically significant. For every additional child born to a participant, there was 28.6% decreased odds of having metabolic syndrome at review (aOR: 0.71, 95% CI 0.40, 1.27; p = 0.25), although also not statistically significant. The odds of having metabolic syndrome at review were more than 15 times higher if metabolic syndrome was present at baseline (aOR: 15.85, 95% CI 3.99, 63.09; p < 0.001).

## Discussion

This study aimed to observe the change in metabolic syndrome status between two time points in a cohort of women referred to the postpartum lifestyle clinic. Overall, we saw a modest reduction of 4.7% in the percentage of the cohort with metabolic syndrome at review compared to baseline, but this was not statistically significant. There were also small but not statistically significant improvements at time of review for the mean values of peripheral systolic blood pressure, triglycerides, HDL cholesterol, glucose, and insulin, and the number of current smokers reduced by one participant (16.7%). Although mean BMI slightly increased from baseline to review, this was not reflected in the waist circumference measurements, which slightly reduced.

Over half of the study cohort (57.8%) did not have metabolic syndrome at either time point, which is promising for future cardiometabolic health if this persists long-term. However, 91.6% and 89.1% of the entire cohort had at least one criterion for metabolic syndrome at baseline and review, respectively. Of the patients with metabolic syndrome at baseline, 8 (36%) recovered and did not meet the criteria at review. There was an overall mean improvement in all of the cardiometabolic risk factors, with the most notable change being observed in the mean fasting insulin levels (Table 3). This could indicate that these participants may have benefited from the postpartum lifestyle clinic and taken steps to reduce their cardiometabolic risk. However, 5 (7.8%) participants did not have metabolic syndrome at baseline but did meet the criteria at review. Over 20% of the cohort had metabolic syndrome at both time points. It is possible that the intervention may not have been effective for these participants, or perhaps that it was not effective within the study timeframe. The number of participants medicated for hypertension or type 2 diabetes increased from baseline by 1 and 2 participants, respectively. This may suggest an important role of the postpartum lifestyle clinic for identifying patients who need medical therapy after typical postpartum follow-up with general practitioners has ceased.

The percentage of metabolic syndrome at both time points is higher than that reported in other similar studies. A preliminary analysis of our registry cohort at 7 months postpartum found 36% of women had metabolic syndrome [25], higher than the rate reported at baseline in the present study. In a recent comparison of Maternal Health Clinic data with data from a similar clinic in rural Canada, metabolic syndrome prevalence at 6 months postpartum was 21.8% and 29.2%, respectively [34]. These values are lower than the metabolic syndrome prevalence reported at baseline in the current study (34.4%), which may be attributed to the high levels of socioeconomic disadvantage in the area in which our postpartum lifestyle clinic operates. This may also be a reflection of including only medicated or severe cases of the complications of pregnancy in the postpartum lifestyle clinic.

In general, lifestyle interventions have been found to be effective at reducing metabolic syndrome and other cardiometabolic risk factors. A study of adults with metabolic syndrome found that a lifestyle intervention incorporating dietary, exercise and behaviour modification education sessions significantly reduced metabolic syndrome by 72%, central obesity by 67%, the incidence of diabetes by 77% and hypertriglyceridemia by 52% after 12 months [35]. It is important to note, however, that this included a relatively intensive intervention with multiple individual and group sessions, compared with general practitioner advice. The study also focused on middle-aged adults, and not on new mothers. Analyses of the long-term effectiveness of lifestyle interventions, specifically those delivered in the postpartum period, at reducing cardiometabolic disease have not yet been able to be performed due to the length of time required for follow-up. Preliminary results reported in the literature are varied, but positive. A systematic review found that lifestyle interventions of exercise, dietary habits, and smoking cessation are associated with an estimated 4–13% decreased cardiovascular risk after preeclampsia [36]. However, this 2013 review included studies that are now ≥ 15 years old, and cardiovascular risk in almost every study was determined using risk prediction models based on cohorts of mostly older men. The length of follow-up of the studies was also far less than the time span of the cardiovascular risk prediction models [36]. A systematic review of the effectiveness of both technology-based and face-to-face lifestyle interventions at preventing type 2 diabetes in women with a history of gestational diabetes reported that most interventions improved insulin resistance-related and weight-related measures in the short-term, but indicated the long-term effectiveness of these interventions needs further research [37].

Currently, the participants in our cohort receive face-to-face counselling appointments at the clinic at approximately 6 months and eighteen months, with a plan to also review them at 5 years postpartum, with no contact between appointments. Participants also have the option to be referred through the clinic to a free telephone counselling service that provides ongoing dietary and exercise advice. A focus group study of women with a history of preeclampsia, gestational diabetes, or intrauterine growth restriction reported that women found adopting a healthy lifestyle after delivery extremely difficult and that they lacked professional support and guidance to facilitate lifestyle change [38]. It is possible that we need to increase the level of support provided to our participants to see more marked effectiveness. A study by Berks and colleagues found improved cardiometabolic risk factors over seven months of an intervention involving multiple individual counselling sessions and a computer health education program [39]. Furthermore, a dietitian-led intervention for women with gestational diabetes commencing during pregnancy and continuing until seven months postpartum and focusing on diet, exercise, and breastfeeding practices showed some improvements in weight reduction, although this was not statistically significant [40]. Further qualitative research is warranted to identify the ideal frequency and mode of contact with the postpartum lifestyle clinic to maximise effectiveness.

### Limitations

There are a number of limitations in the present study that warrant discussion. This was a preliminary analysis of an ongoing study, and therefore the study was limited by a small sample size. Multinomial logistic regressions to examine the effects of age and parity on the change of metabolic syndrome status over time were not able to be performed due to the small study size. However, preliminary analyses are necessary to establish a baseline of expected results and allows for optimisation of methods if needed. Metabolic syndrome status prior to pregnancy was not able to be assessed, and so it is possible that metabolic syndrome had persisted in some women for a long period of time and pre-dated their pregnancy complication. There was no control group that had not received the intervention, so conclusions about the true effectiveness of the intervention are not able to be made. However, the results of this study provide rationale and preliminary data for conducting a fully powered randomised controlled trial to assess the intervention’s effectiveness at reducing metabolic syndrome and overall cardiometabolic risk.

Our study is inherently limited by self-selection bias, as one-third of the participants who attended a baseline appointment did not attend a review appointment. This is likely to be a result of a combination of the following factors: (1) general loss of follow-up due to address and phone number changes, (2) administration errors, (3) attendance hesitancy due to COVID-19 pandemic, (4) inability to attend an appointment on the designated clinic days and times, or due to time constraints. Further research exploring the barriers to attendance are required. No participants referred to the clinic for preterm labour or placental abruption were eligible to be included in this study, and there was a high rate of gestational diabetes in the cohort. It is likely that future analyses from this ongoing study will be able to incorporate a wider range of participants and those with the rarer complications of pregnancy, such as spontaneous preterm labour and placental abruption.

Finally, we did not assess the effectiveness of the intervention on the outcomes of any subsequent pregnancies. A recent retrospective analysis of more than 66,000 singleton pregnancies found that achieving optimal weight gain during pregnancy significantly reduced development of late-onset preeclampsia in overweight or obese women (OR 0.42, p < 0.0001) [41], supporting the potential role of lifestyle intervention prior to or during pregnancy to assist in weight management. A recent study of women with an index pregnancy complicated by gestational diabetes or a hypertensive disorder found that attendance at the Maternal Health Clinic was associated with inter-pregnancy weight reduction, fewer inter-pregnancy diagnoses of type 2 diabetes, and delivery of subsequent pregnancy at a later gestational age [14]. A meta-analysis of lifestyle interventions delivered during pregnancy also found that dietary interventions reduced the risk of preeclampsia by 33% when compared to controls [42]. However, a more recent randomised control trial of healthy weight women found that lifestyle intervention had no impact on development of any maternal complications of pregnancy [43]. Another study of overweight or obese women recruited early in pregnancy found that diet and exercise advice had no impact on (1) cardiometabolic inflammatory markers, including glucose, HDL cholesterol, triglycerides, and insulin, at multiple time points during pregnancy [44], and (2) development of maternal complications of pregnancy [45]. Future research exploring the effect, if any, of our postpartum lifestyle clinic on subsequent pregnancies should be conducted.

## Conclusion

The results of this preliminary analysis confirmed a high rate of metabolic syndrome in an early postpartum cohort of women who had recently experienced a complication of pregnancy. The study showed a modest improvement in the overall prevalence of metabolic syndrome at review compared to baseline. There were also some minor improvements in individual cardiometabolic risk factors over time. These results highlight the importance of continuing preventative care for this high-risk group of women. Future research will examine the effectiveness of our nurse practitioner-led lifestyle intervention at reducing metabolic syndrome through a fully powered randomised controlled trial.

## Data Availability

The datasets generated and/or analysed during the current study are not publicly available due local ethical regulations and privacy principles but are available from the corresponding author on reasonable request and with permission of local ethics committees.
